# Parathyroid hormone-related protein inhibits nitrogen-containing bisphosphonate-induced apoptosis of human periodontal ligament fibroblasts by activating MKP1 phosphatase

**DOI:** 10.1080/21655979.2021.1928930

**Published:** 2021-05-23

**Authors:** Di Liu, Juan Du, Jing Sun, Minqi Li

**Affiliations:** a Department of Bone Metabolism, School and Hospital of Stomatology, Cheeloo College of Medicine, Shandong University & Shandong Key Laboratory of Oral Tissue Regeneration & Shandong Engineering Laboratory for Dental Materials and Oral Tissue Regeneration, Jinan China; b Department of Prosthodontics, School and Hospital of Stomatology, Cheeloo College of Medicine, Shandong University & Shandong Key Laboratory of Oral Tissue Regeneration & Shandong Engineering Laboratory for Dental Materials and Oral Tissue Regeneration, Jinan China; c Department of Oral and Maxillofacial Surgery, Shandong Provincial Hospital Affiliated to Shandong First Medical University, Jinan China; d Department of Periodontology, Jinan Stomatological Hospital, Jinan China

**Keywords:** Parathyroid hormone-related protein, human periodontal ligament fibroblasts, nitrogen-containing bisphosphonates, mapk phosphatase-1

## Abstract

Massive production of reactive oxygen species (ROS) in human periodontal ligament fibroblasts (HPdLFs) by nitrogen-containing bisphosphonates (BPs) is the main factor causing BP-related osteonecrosis of the jaw. Further, oxidative stress and apoptosis of fibroblasts induced by ROS are closely associated with the activation of MAPK. Parathyroid hormone-related protein (PTHrP) can block the activity of MAPK by regulating the levels of MAPK phosphatase 1 (MKP1). Therefore, it is speculated that PTHrP can inhibit the apoptosis of HPdLFs caused by nitrogen-containing BP via regulating the expression levels of MKP1. Herein, alendronate sodium salt trihydrate (nitrogen-containing BP, FOS) and HPdLFs were co-cultured for 24 h, 48 h, and 72 h, and the levels of ROS and apoptosis were determined, respectively. After 48 h co-culture, FOS significantly increased the levels of ROS and apoptosis, and high phosphorylation levels of p38, ERK1/2 and p66^Shc^ were found in this study. However, the inhibitors of p38 and ERK1/2 significantly reduced the apoptosis of HPdLFs. Interestingly, PTHrP pre-treatment significantly reduced the phosphorylation levels of p38, ERK1/2, and p66^Shc^. More importantly, MKP1 inhibitor sanguinarine inhibited the dephosphorylation levels of p38, ERK1/2, and p66^Shc^ caused by PTHrP. Altogether, PTHrP can inhibit nitrogen-containing BP-induced apoptosis of HPdLFs by activating MKP1 phosphatase.

## Graphical abstract

**Figure:**
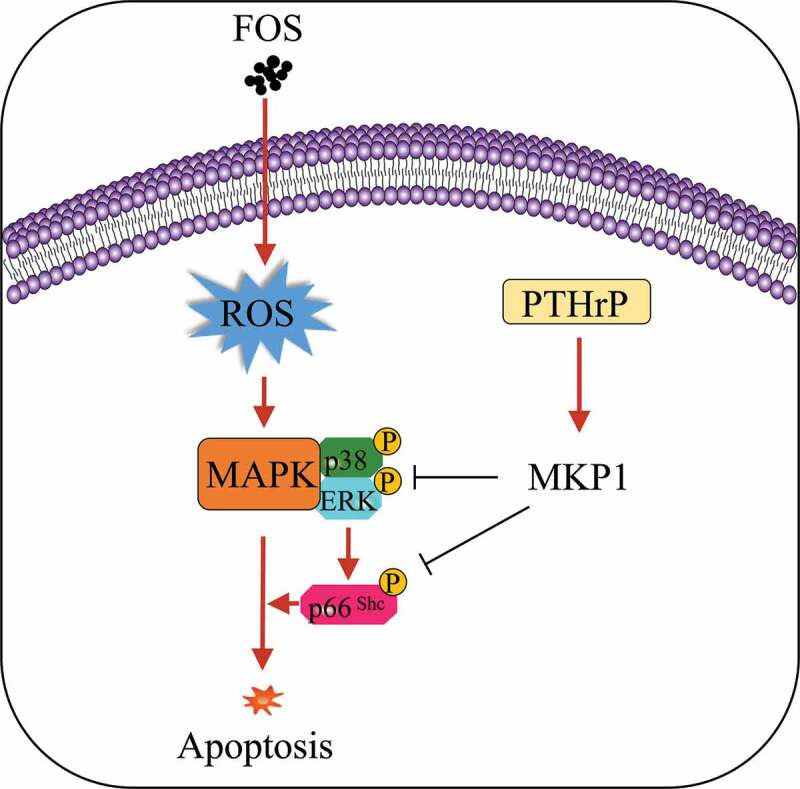

## 1. Introduction

Bisphosphonates (BPs), which functionally serve as bone resorption inhibitors, have been used in medicine for nearly 30 years, and play an important role in the treatment of malignant bone tumors (multiple myeloma and bone metastasis) or metabolic bone diseases (Paget’s disease and severe osteoporosis) [1]. BPs are divided into first, second, and third generations, among which the second and third generations of BPs containing nitrogen exhibit a stronger inhibitory effect on bone resorption [2,3]. Nitrogen-carrying BPs such as alendronate sodium salt trihydrate (FOS) and zoledronate acid (ZOL), play an important role in the treatment of osteoporosis and bone metastasis [3-6]. However, in recent years, with an increasing use of nitrogen-carrying BPs, the number of cases of nitrogen-carrying BP-related osteonecrosis of the jaw (BRONJ) is gradually increasing, which is mainly related with the fact that nitrogen-carrying BPs can cause strong oxidative stress and cellular apoptosis through the overproduction of reactive oxygen species (ROS) [7,8].

ROS are produced during oxidative phosphorylation of the mitochondria or may be produced by exogenous substances, such as xenobiotic compounds [9]. When the ROS content exceeds the antioxidant defense capacity of cells, it can damage the nucleic acids, proteins, and lipids in cells, causing some clinical symptoms [10,11]. Pharmacological and genetic studies on mice have confirmed the harmful effects of oxidative stress on bones [10]. The harmful effects of oxidative stress involve various molecular mechanisms of cellular processes, including apoptosis and aging [11,12]. ROS can cause the reversible oxidation of cysteine residues, resulting in auto-phosphorylation of proteins, such as apoptosis signal-regulated kinases (ASKs), and activation of downstream mitogen-activated protein kinases (MAPKs) p38, ERK1/2, jun N-terminal kinases (JNKs), and phosphatidylinositol 3-kinase (PI3K)/Akt [13-15]. Cellular stress stimulation, as observed during hypoxia and mechanical and oxidative challenges, can regulate the apoptosis of many cell types, such as osteoblasts [12,16], cardiomyocytes [17], fibroblasts [18], and periodic ligament stem cells [19], by activating the above mentioned kinases. In many cell types, such as osteoblasts [20] and marginal cells [21], p66^Shc^, an amplifier of ROS, is an important mediator for ROS-induced apoptosis.

Hormones such as thyroid hormones and melatonin, which are extracellular regulators of oxidative stress, can target various signaling pathways in a variety of tissues [10,20,22,23]. Parathyroid hormone (PTH) and its bone counterpart PTH-related protein (PTHrP) combine with the common PTH type 1 receptor (PTH1R), which can increase the survival rate of cells by regulating intracellular signaling pathways such as ERK1/2 and p38MAPKs, thus promoting partial bone formation [24-27]. MAPK phosphatase-1 (MKP1) belongs to the threonine-tyrosine phosphatase family, which can inhibit the activity of ERK, p38MAPKs, and JNK through dephosphorylation [28,29]. Previous studies have indicated that PTHrP can inhibit the activity of MAPKs by regulating the expression of MKP1, and thus inhibit the apoptosis of osteoblasts [30].

In view of the fact that the oxidative stress and apoptosis of fibroblasts induced by ROS are closely associated with the activation of MAPK and that PTHrP can regulate the activity of MAPK by regulating MKP1, it can be speculated that PTHrP can inhibit ROS-induced apoptosis of fibroblasts by regulating the expression of MKP1. To verify this hypothesis, the levels of ROS and apoptosis were determined after FOS and HPdLFs were co-cultured, and the relationship between PTHrP and the expression level of MKP1 was comprehensively analyzed to disentangle whether and how PTHrP pre-treatment inhibits FOS-induced apoptosis of HPdLFs at the molecular level.

## 2. Materials and methods

### 2.1 Reagents and antibodies

Nitrogen-containing BP, alendronate sodium salt trihydrate (FOS) was purchased from Wako in Japan. SCGMTM BulletKitTM, N-acetyl-cysteine (NAC), fluorescein isothiocyanate (FITC)-conjugated annexin V, and ECL chemiluminescence were acquired from Sigma in China. PTHrP, cell-permeable 5-(and-6)-chloromethyl-2′, 7′-dichlorodihydrofluorescein diacetate, acetyl ester (CM-H2DCFDA), U0126 (inhibitor of ERK1/2), SB203580 (inhibitor of p38), sanguinarine chloride (inhibitor of MKP1), lipofectamine LTX transfection reagent, Tris HCl, NaCl, EDTA, 1% Triton X-100, 1% sodium hydroxide, 0.1% sodium dodecyl sulfate (SDS), protease inhibitor cocktail, cocktail Set II, bovine serum albumin (BSA), Considering bicinchoninic acid (BCA), nitrocellulose membrane were obtained from ThermoFisher in China. In addition, α-Tubulin, peroxidase-conjugated goat anti-rabbit IgG and the antibodies of p-ERK1/2, ERK1/2, p-Akt, Akt, p-p38, p38, p-JNK, JNK, MKP-1, and p-p66^Shc^ (Ser36) were acquired from ThermoFisher in China. Propidium iodide was purchased from Solarbio in China.

### 2.2. Cell culture condition and experimental design

The normal adult HPdLFs used in this study were purchased from ScienCell (USA). The HPdLFs were cultured with SCGMTM BulletKitTM (Sigma, China) at 37°C with 5% CO_2_.

To investigate the effect of FOS on HPdLFs, after the HPdLFs were treated with 30 µM FOS for 24 h, 48 h, and 72 h, the levels of ROS and apoptosis were determined. Based on the result, HPdLFs treated with 30 µM FOS for 48 h were selected as experimental subject, and then the phosphorylation levels of p38, ERK1/2, Akt, JNK and p66^Shc^ were analyzed. In addition, the inhibitors of p38 (SB203580 [5 µM]) and ERK1/2 (U0126 [10 µM]) were used to analyze the roles of p38 and ERK1/2 in apoptosis of HPdLFs.

To disentangle whether and how PTHrP inhibits FOS-induced apoptosis of HPdLFs, following the treatment with 30 µM FOS, the HPdLFs were treated with 100 nM PTHrP for 30 min, and then with sanguinarine chloride [0.5 µM] for 1 h to analyze the relationship between PTHrP and the expression level of MKP1.

### 2.3 Determination of ROS

The HPdLFs (1 × 10^3^) were incubated in a 96-well plate for 24 h, and then treated with 30 µM FOS for 24 h, 48 h, and 72 h, respectively. The cells untreated with FOS in the corresponding time points were considered as controls. Approximately 12 h before the cells were treated with FOS, 90 mM NAC was added to the cell suspension [7,31]. To determine the ROS content, CM-H2DCFDA was added to the culture medium until the final concentration was 10 µM. The fluorescence was determined using a fluorescence spectrophotometer (Infinite M200 Pro) at 488 nm excitation and 525 nm emission.

### 2.5. Detection of apoptosis by flow cytometry

To measure apoptosis, the cells were double-stained with fluorescein isothiocyanate (FITC)-conjugated annexin V and propidium iodide for 5 min at room temperature. The number of annexinV(-)/propidium iodide(-) living cells and annexinV(+) apoptotic cells was examined by flow cytometry (ThermoFisher, USA). The data were collected with FACSCalibur^TM^ (Becton Dickinson) and analyzed with CellQuest^TM^ (Becton Dickinson).

### 2.6. Western blot analysis

Total cellular protein was extracted at pH 7.4 with 50 mM Tris HCl, 150 mM NaCl, 1 mM EDTA, 1% Triton X-100, 1% sodium hydroxide, 0.1% sodium dodecyl sulfate (SDS), protease inhibitor cocktail, and cocktail Set II. Considering bovine serum albumin (BSA) as the standard, bicinchoninic acid (BCA) was used to determine the content of protein in the cellular extract. The proteins were isolated using 5%-10% polyacrylamide-SDS gels (30–60 μg/Lane). After electrophoresis, the sample was transferred to a nitrocellulose membrane, and blocked with 5% skim milk containing 50 mmol/L Tris-HCl (pH 7.5) and 150 mmol/L NaCl containing 0.05% Tween 20. The corresponding antibodies p-ERK 1/2 (1:1000), ERK 1/2 (1:2000), p-Akt (1:1000), Akt (1:2000), p-p38 (1:1000), p38 (1:2000), p-JNK (1:1000), JNK (1:2000), MKP-1 (1:500), and p-p66^Shc^ (Ser36) (1:500) were added, followed by incubation overnight at 4°C. α-Tubulin was used as the loading control. After washing, peroxidase-conjugated goat anti-rabbit IgG was incubated with the membrane, and ECL chemiluminescence was added. The optical density of the protein band was normalized to the corresponding α-tubulin band.

### 2.7. Statistical analysis

The data are expressed as mean±standard error of mean (SEM). Nonparametric variance analysis (Kruskal–Wallis) was used to evaluate changes in kinase phosphorylation and apoptosis, followed by post-hoc Dunn’s test. *P* < 0.05 was considered significant.

## 3. Results

### 3.1. FOS induces the apoptosis of HPdLFs by regulating the generation of ROS

**Figure 1.**
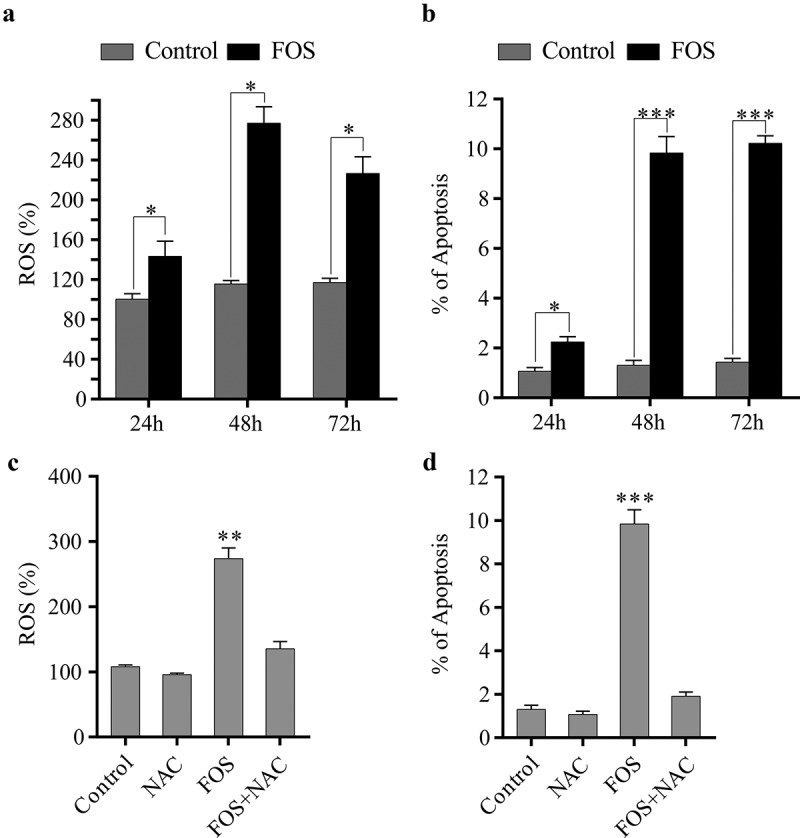
ROS generation by FOS treatment induced cell apoptosis in HPdLFs. (a) FOS increased ROS production in HPdLFs after 24, 48, and 72 h. (b) FOS increased apoptosis in HPdLFs after 24, 48, and 72 h. (c) NAC suppressed FOS-induced ROS generation in HPdLF after 48 h. (d) NAC suppressed FOS-induced apoptosis in HPdLF after 48 h. Data represented mean±standard error of mean (SEM) of six independent experiments. *P < 0.05, **P < 0.01 and ***P < 0.001 versus control

Previous studies have shown that BPs can induce the generation of ROS in HPdLFs. To verify this conclusion, HPdLFs were treated with 30 μM of FOS for different time points (24 h, 48 h, and 72 h). Subsequently, the ROS content was determined and it was found that the ROS content was significantly increased after FOS and HPdLFs were co-cultured for 48 h (Figure 1a). Previous studies have shown that ROS are the main factor causing cellular apoptosis. Therefore, the degree of apoptosis of HPdLFs was examined, and we found that the degree of apoptosis was significantly increased after FOS and HPdLFs were co-cultured for 48 h (Figure 1b). To further study whether the apoptosis of HPdLFs induced by FOS was associated with ROS, after HPdLFs were co-cultured with FOS and the ROS scavenger NAC for 48 h, and the level of ROS and the degree of apoptosis were subsequently determined. It was found that NAC can significantly reduce the level of ROS and the degree of apoptosis (Figure 1c, d), indicating that FOS induced the apoptosis of HPdLFs by promoting the generation of ROS.

### 3.2. The role of different protein kinases in FOS-induced apoptosis of HPdLFs

**Figure 2.**
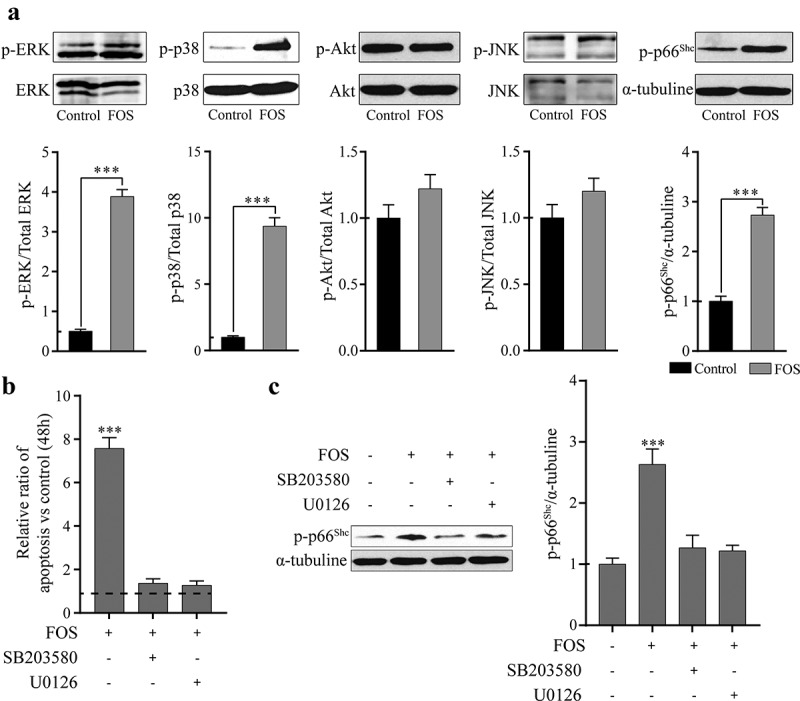
FOS induced apoptosis by triggering phosphorylation of p38, ERK1/2, and p66^Shc^ in HPdLFs. (a) FOS (30 μM) significantly increased ERK1/2 (44/42 KDa), p38 (43 KDa), and p66^Shc^ (66 KDa) (pro-apoptotic and oxidative marker) phosphorylation, at 48 h in HPdLFs. (b) FOS-induced apoptosis was inhibited by pretreatment with p38 (5 mM SB203580) or ERK1/2 (10 mM U0126) inhibitors for 30 min in HPdLFs. (c) FOS-induced phosphorylation of the oxidative stress and apoptosis marker p66^Shc^ was suppressed by pretreatment with p38 (5 mM SB203580) or ERK1/2 (10 mM U0126) inhibitors for 30 min in HPdLFs. Data represented mean±standard error of mean (SEM) of six independent experiments. ***P < 0.001 versus control

It has been reported that ROS can regulate intracellular kinases and activate the related cell signaling pathways [9,12]. Therefore, to study whether ROS produced by FOS can activate the kinases related to cell activity, the effect of FOS on the phosphorylation of cell survival-related kinases was examined, and it was found that the phosphorylation degrees of ERK1/2, p38MAPK, and the pro-apoptotic and oxidative stress marker p66^Shc^ were significantly increased (Figure 2a), whereas no significant changes were observed in the phosphorylation levels of Akt and JNK. To study whether FOS affects the survival of HPdLFs by regulating the phosphorylation of ERK1/2 and p38, FOS and HPdLFs were co-cultured in a media containing ERK1/2 and p38 inhibitors, respectively, and the degree of cell apoptosis was then determined. We found that ERK1/2 and p38 inhibitors can significantly reduce the apoptosis induced by FOS (Figure 2b). Phosphorylation levels of ERK1/2 and p38 have been reported to affect the activity of p66^Shc^. Thus, we analyzed the effect of ERK1/2 and p38 inhibitors on the phosphorylation of p66^Shc^ and found that ERK1/2 and p38 inhibitors can inhibit p66^Shc^ phosphorylation induced by FOS (Figure 2c). This indicated that FOS could promote the phosphorylation of p66^Shc^ by regulating ERK1/2 and p38, and subsequently induce apoptosis.

### 3.3. PTHrP inhibits the phosphorylation of kinases in an MKP1-dependent manner

**Figure 3.**
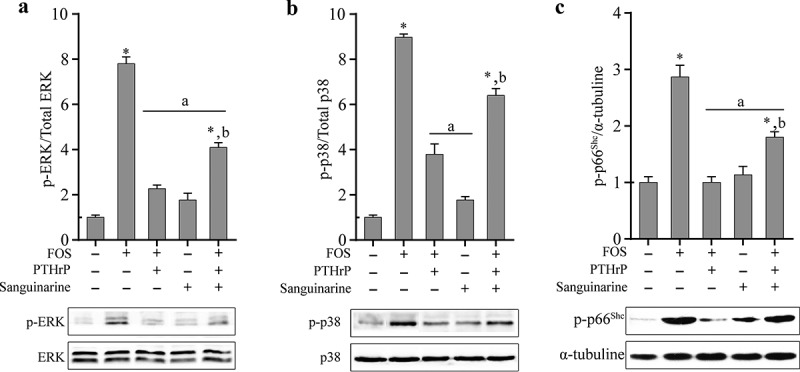
PTHrP inhibited FOS-dependent kinase phosphorylation by mediating MKP1 in HPdLFs. PTHrP reduced FOS-induced ERK1/2 activation in HPdLFs, and the effect was suppressed by MKP1 inhibitor, sanguinarine (0.5 mM) (a). PTHrP reduced FOS-induced p38 activation in HPdLFs, and the effect was suppressed by MKP1 inhibitor, sanguinarine (0.5 mM) (b). PTHrP reduced FOS-induced p66^Shc^ activation in HPdLFs, and the effect were suppressed by MKP1 inhibitor, sanguinarine (0.5 mM) (c). Data represented mean±standard error of mean (SEM) of six independent experiments. *P < 0.05 versus control; ^a^*P*<0.05 versus FOS stimulation; ^b^*P*<0.05 versus corresponding condition without inhibitor

It has been reported that PTHrP can inhibit the phosphorylation of MAPKs induced by H_2_O_2_-induced ROS in osteoblasts [30]. To study whether PTHrP can inhibit the phosphorylation of ROS-induced kinases induced by FOS in HPdLFs, HPdLFs pre-treated with PTHrP were co-cultured with FOS for 48 h, followed by the examination of phosphorylation. It was found that PTHrP can significantly reduce the FOS-induced phosphorylation levels of ERK1/2, p38, and p66^Shc^ (Figure 3). Previous studies have shown that PTHrP can regulate the activities of p38, ERK1/2, and p66^Shc^ through MKP1 [30]. Therefore, we evaluated whether PTHrP inhibits the phosphorylation levels of p38, ERK1/2, and p66^Shc^ induced by FOS through MKP1. To verify this hypothesis, as described previously [32-34], we used MKP1 inhibitor sanguinarine to pre-treat HPdLFs and found that the phosphorylation levels of ERK1/2 and p66^Shc^ in HPdLFs treated with PTHrP and FOS were significantly higher (Figure 3), indicating that MKP1 inhibitor sanguinarine can significantly inhibit the dephosphorylation ability of PTHrP for ERK1/2 and p66^Shc^.

### 3.4. PTHrP inhibits FOS-induced apoptosis by regulating the expression of MKP1

**Figure 4.**
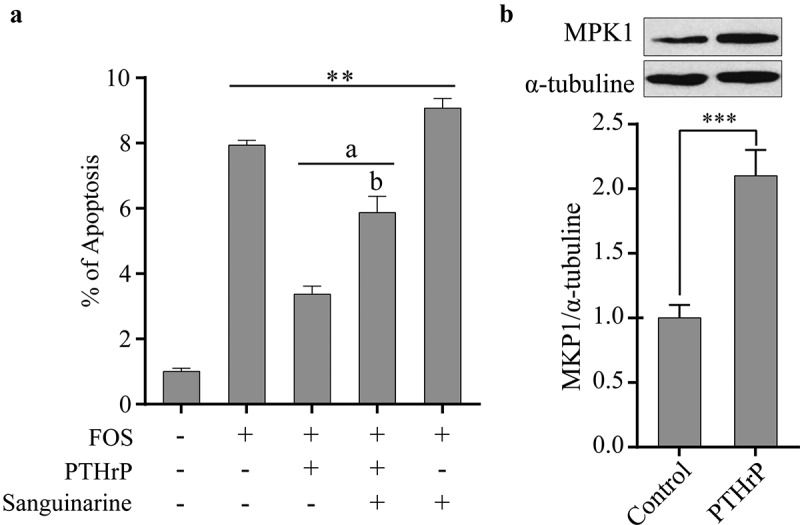
PTHrP inhibited FOS-induced apoptosis by MKP1 in HPdLFs. (a) PTHrP inhibited FOS-induced apoptosis in HPdLFs, and the effect was suppressed by MKP1 inhibitor, sanguinarine, (0.5 mM). (b) PTHrP increased protein level of MKP1 in HPdLFs. Data represented mean±standard error of mean (SEM) of six independent experiments. **P < 0.01 and ***P < 0.001 versus control; ^a^*P*<0.05 versus FOS stimulation; ^b^*P*<0.05 versus the corresponding FOS + PTHrP (without inhibitor)

To determine whether PTHrP can inhibit the apoptosis of HPdLFs induced by FOS, HPdLFs cells were co-cultured with PTHrP and FOS for 48 h and the degree of apoptosis was examined. It was found that PTHrP could significantly inhibit FOS-induced apoptosis of HPdLFs (Figure 4a). However, sanguinarine can completely abolish this inhibitory effect of PTHrP on the apoptosis of HPdLFs (Figure 4a). To investigate whether PTHrP can promote the expression of MKP1 in HPdLFs, HPdLFs were pre-treated with PTHrP for 30 min, and the protein level of MKP1was then determined. We found that PTHrP can promote the expression of MKP1 (Figure 4b). The above mentioned results indicated that PTHrP can inhibit the phosphorylation levels of p38 and ERK1/2 by promoting the expression of MKP1, thus inhibiting the apoptosis of HPdLFs induced by FOS.

## 4. Discussion

The massive production of ROS can damage HPdLFs, and subsequently cause BRONJ [7]. During the process of cellular response to oxidative damage, MAPKs, such as ERK1/2, p38, and JNK, can be activated, and can eventually affect the survival, proliferation, and differentiation ability of various types of cells, such as cardiomyocytes [17], hepatocytes [35], T lymphocytes [36], osteoblasts [12,16], and fibroblasts [18]. Previous studies have shown that PTHrP can inhibit the activation of MAPKs induced by ROS [37]. Therefore, we hypothesized that PTHrP can inhibit the pro-apoptotic effect of ROS on HPdLFs by regulating the MAPK-signaling pathway. Our results indicated that PTHrP can inhibit the FOS-induced apoptosis of HPdLFs by activating MKP1.

It has been reported that BPs can induce the generation of ROS in many types of cells, such as human gingival fibroblasts [38], osteoclast precursor cells [39], and mature osteoclast-like cells [39]. In this study, after FOS and HPdLFs were co-cultured for 48 h, we found that the level of ROS in HPdLFs and the degree of HPdLF apoptosis both significantly increased. Previous studies have shown that ROS can induce the apoptosis of many types of cells, such as osteoblasts [10] and human embryonic fibroblasts [40], by activating MAPKs (such as ERK1/2, JNK, p38, and Akt). This study indicated that ERK1/2 and p38 were activated by FOS in HPdLFs. In some cell lines, the activated ERK can induce the release of mitochondrial cytochrome c, the activation of caspase-8, permanent cell cycle arrest, or the formation of autophagic vacuoles [41]. Under normal condition, the redox regulatory protein thioredoxin (Trx) binds to and inhibits the activity of the protein apoptosis signal-regulated kinase 1 (ASK1), which is associated with p38 and JNK activation. However, ROS can cause the dissociation of the Trx-ASK1 complex, which in turn activates p38 and JNK and triggers apoptosis [12,42]. Although the activation of JNK and Akt induced by ROS plays an important role in the regulation of apoptosis, our results show that FOS cannot activate JNK and Akt [43]. Therefore, JNK and Akt may not be involved in the FOS-mediated apoptosis pathway of HPdLFs.

PTHrP regulates various biological processes of various mammalian cells, such as cell differentiation, proliferation, and apoptosis [44,45]. Previous studies have shown that PTHrP can inhibit H_2_O_2_-induced apoptosis of osteoblasts by regulating the phosphorylation of p38 MAPKs and ERK [30]. This study showed that exogenous PTHrP can inhibit the phosphorylation of p38 MAPK and ERK and FOS-induced apoptosis of HPdLFs, indicating that PTHrP can inhibit FOS-induced apoptosis of HPdLFs by regulating p38 MAPK and ERK signaling pathway. In addition, PTHrP can inhibit the phosphorylation of ERK and p38 by promoting the expression of MKP1, thereby inhibiting the apoptosis of osteoblasts induced by H_2_O_2_ [30]. This study indicated that the protein level of MKP1 in HPdLFs pre-treated with PTHrP was significantly increased, and that sanguinarine an inhibitor of MKP1 can significantly reduce the dephosphorylation of p38 MAPK and ERK1/2 induced by PTHrP. Further, we found that PTHrP can inhibit the apoptosis of HPdLFs by promoting the expression of MKP1 and inhibiting the phosphorylation of p38 and ERK1/2 induced by FOS. It is worth noting that sanguinarine completely inhibited the dephosphorylation of PTHrP to p38, and partially inhibited the dephosphorylation of PTHrP to ERK. MKP1 has been reported to dephosphorylate MAPK family proteins according to affinity (p38 ≥ JNK > > ERK1/2) [46]. Therefore, the expression of MKP1 induced by PTHrP may completely inhibit the activity of p38 in HPdLFs, but it cannot completely inhibit the activity of ERK.

It has been reported that the phosphorylation of p66^Shc^ at Ser36 can regulate oxidative stress-related apoptotic response and cell senescence [47]. The phosphorylation of p66^Shc^ can open the mitochondrial permeability transition pore, leading to the swelling of organelles [48]. Subsequently, the disruption of mitochondrial integrity can lead to the release of a variety of pro-apoptotic mitochondrial factors into the cytosol, thus activating the apoptotic cascade pathway and causing cell death [49]. Phosphorylated p66^Shc^ can not only regulate apoptotic signals but also continuously enhance the production of ROS through different mechanisms. Therefore, regulation of p66^Shc^ phosphorylation may be the key target for inhibiting apoptosis induced by ROS. Our results show that a part of dephosphorylation of PTHrP to p66^Shc^ is regulated by MKP1 in HPdLFs. In response to the process of oxidative stress, the activation of ERK1/2 and p38 may promote the phosphorylation of p66^Shc^ at Ser36, Ser54, and Thr386 [50,51], indicating that phosphorylated p66^Shc^ may be a downstream effector in the ERK1/2 and p38 pro-apoptosis process. We found that the inhibitors of ERK1/2 and p38 can inhibit the FOS-induced phosphorylation of p66^Shc^, suggesting that p66^Shc^ is a downstream regulator of ERK1/2 and p38 during the process of FOS-induced HPdLF apoptosis.

## 5. Conclusion

In summary, our results suggest that PTHrP inhibits nitrogen-containing BP-induced apoptosis of HPdLFs by activating MKP1 phosphatase.

## Highlights

FOS-induced ROS cause oxidative stress and cellular apoptosis in HPdLFs

Cellular apoptosis of HPdLFs is closely associated with the activation of MAPK

PTHrP can regulate the activity of MAPK by regulating the levels of MKP1
